# Food oral immunotherapy

**DOI:** 10.1186/s13223-025-00948-5

**Published:** 2025-02-12

**Authors:** Mary McHenry, Philippe Bégin, Edmond S. Chan, Meriem Latrous, Harold Kim

**Affiliations:** 1 Pediatric Allergy & Clinical Immunology, Dalhousie University/IWK Health Centre, Halifax, NS Canada; 2 Division of Clinical Immunology and Allergy, Department of Medicine, Université de Montréal, Montréal, Québec Canada; 3 Division of Allergy, Department of Pediatrics, University of British Columbia, British Columbia Children’s Hospital, Vancouver, BC Canada; 4 Division of Infectious Diseases, Immunology, and Allergy, Department of Pediatrics, Children’s Hospital of Eastern Ontario, Ottawa, ON Canada; 5 Division of Clinical Immunology & Allergy, Department of Medicine, McMaster University, Hamilton, ON Canada; 6 Division of Clinical Immunology and Allergy, Department of Medicine, Western University, London, ON Canada

## Abstract

Food oral immunotherapy (OIT) is an option for the treatment of immunoglobin E (IgE)‐mediated food allergy that involves administering gradually increasing doses of an allergenic food over time (under medical supervision) with the goal of desensitizing an individual to the food allergen. Current Canadian clinical practice guidelines for OIT recommend this form of therapy as an option in patients with food allergy. The intervention should be prioritized in the infant and toddler population, in which it is particularly well tolerated and can lead to sustained unresponsiveness (also sometimes referred to as remission). In this article, we provide an overview of OIT and discuss the role non-allergist clinicians can play in caring for patients undergoing OIT.

## Key take-home messages

- OIT is a safe and effective treatment option for IgE-mediated food allergy.
- Early OIT, especially during infancy and preschool age, appears to be safer and more effective compared to starting OIT in older children.
- OIT is disease-modifying when started early. It should therefore be provided as a management option for families of infants who have failed primary prevention of food allergy. Referral to an allergist who offers this form of therapy should be considered.
- Choosing OIT as a treatment option involves shared decision-making between the patient, family, and allergist. Goals of treatment are important to establish at the start of treatment.
- Patients in clinical trials undergoing OIT may experience more allergic reactions than those treated with placebo, but reactions tend to decrease in frequency and severity as treatment progresses. The risk of reactions with avoidance in the real world may be as high as with OIT.
- For higher-risk patients, a safer approach is an initial phase of SLIT to bypass OIT build-up.
- Non-allergist clinicians (i.e., family physicians, pediatricians) can play an important role in identifying patients who may benefit from OIT. They can also be instrumental in helping patients undergoing OIT prevent and manage possible adverse reactions during therapy.

## Introduction

Food oral immunotherapy (OIT) is an approach to the treatment of patients with immunoglobin E (IgE)‐mediated food allergy (see *IgE-mediated Food Allergy* article in this supplement) [1]. It consists of daily ingestion of the offending food allergen (food dosing), starting below a patient’s threshold dose (i.e., the minimum amount of food protein that would elicit an allergic reaction), and increasing the dose over time with a goal of increasing clinical tolerance to that food [2]. For infants with a diagnosis of new food allergy, OIT should be discussed as a treatment option, and if it is not offered by the patient’s allergist, referral to an allergist who provides this form of therapy should be considered. OIT may also be an option for older children and adults as part of their food allergy management. Recently published Canadian clinical practice guidelines for OIT favorably recommend the use of OIT outside the research setting [2]. Although these guidelines also suggest that OIT may be an option for adults with food allergy, it is not currently offered to adults by the majority of Canadian allergists. This article will define key terms and concepts related to OIT as well as review the safety and efficacy of this form of therapy and the role of non-allergist clinicians in managing patients on OIT.

### Key concepts and definitions related to OIT

**Fig. 1:**
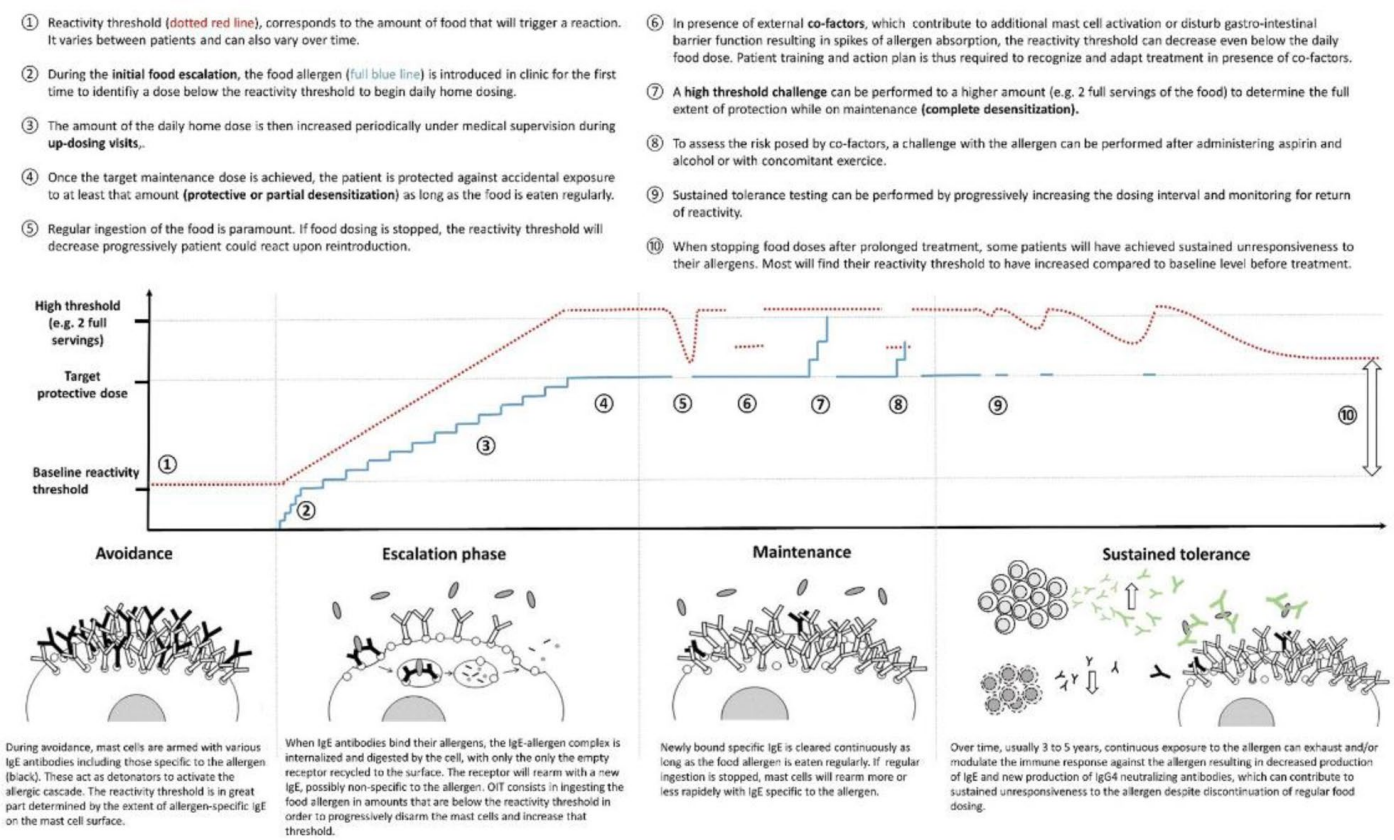
Typical phases of an OIT protocol. Reproduced from Bégin et al. 2020 [2]. Creative Commons license: https://creativecommons.org/licenses/by/4.0/ No changes have been made to the figure

Definitions of key terms and concepts related to OIT are provided in Table 1 and Fig. 1. The initial goal of OIT is clinical desensitization, which is defined as an increase in the threshold of allergen required to cause an allergic reaction while on therapy (i.e., with regular ingestion of the allergen) [3]. Longer term goals include sustained unresponsiveness (also sometimes referred to as remission) and, ideally, oral or immunological tolerance. Sustained unresponsiveness is defined as a state in which the patient who is desensitized can stop eating the food for a period of time after OIT has been discontinued and remains non-reactive when the food allergen is re-ingested. Underlying immune mechanisms for desensitization and sustained unresponsiveness are illustrated and described at the bottom of Fig. 1.

**Table 1:** Definition of key terms related to OIT

Term	Definition
Sensitization	The presence of allergen-specific IgE, or reactivity to an allergen in a skin prick test or serum specific IgE (blood test)
Clinical reactivity	Symptoms of an allergic reaction following allergen ingestion
Desensitization	Increase in allergen consumption threshold in allergic patients while on therapy
Sustained unresponsiveness (remission)	Absence of clinical reactivity to an ingested food allergen at some point (e.g., weeks or months) after therapy has been discontinued and allergen has been avoided
Oral tolerance (immunological tolerance)	Typical immune response to ingested food antigens in healthy individuals that allows for ingestion of foods without adverse reactions, regardless of the length of time since food consumption

IgE: immunoglobulin E

Adapted from Phelps 2022 [3]

Figure 1 illustrates the typical phases of an OIT protocol. Generally, the first step of OIT involves an initial rapid dose-escalation phase where the food allergen is introduced under medical supervision to identify a dose below the reactivity threshold to begin daily home dosing. The amount of the daily home dose is then increased every few weeks during medically supervised up-dosing visits (i.e., build-up phase) until a predefined target maintenance dose is reached. During the maintenance phase, home OIT dosing continues daily at the same dose to: (1) maintain desensitization, and (2) to induce sustained changes in the immune response to the allergen over time. Patients generally remain on their maintenance dose for a prolonged period with follow-up clinic visits to monitor for safety, assess immunologic response and/or protection on and off therapy with oral food challenges (OFCs) (a procedure in which the patient eats the suspected allergenic food in incrementally increasing doses, up to a serving size, under close medical supervision).

Immune changes with OIT are expected to plateau over 3–5 years. These include a decrease in T helper 2 (T_H_2) responses and increased regulatory T cell responses, resulting in an initial increase followed by a decrease in allergen-specific IgE and the production of allergen-neutralizing immunoglobulin G4 (IgG4) antibodies [2]. Sustained benefit in infants and toddlers is likely driven by the decrease in specific IgE levels which tend to decrease below pre-OIT levels, as well as increased IgG4. In older patients, sustained benefits are likely mostly driven by the increase in IgG4 since specific IgE tend not to decrease below pre-OIT levels. Based on patient objectives and preferences, some may wish to continue regular dosing indefinitely to retain full protection. Another option is to discontinue daily dosing for a period of time (e.g., weeks or months) and perform an OFC in clinic to assess the extent of sustained protection off therapy.

### Benefits of OIT

Several clinical trials and meta-analyses have found substantial benefits for patients undergoing OIT [4–9]. Desensitization rates reported in OIT studies, including phase 3 clinical trials, generally range between 67 and 92%, and depend on the definition used (e.g., tolerance to 1 vs. 16 peanuts) and the patient population studied [6–9]. While desensitized, the patient is protected against accidental exposures with amounts below their new reactivity threshold. In some cases, it may allow the patient to introduce the allergen in their regular diet. In practice, desensitization even against small amounts can translate into a significant impact on quality of life (QoL) for patients. Compared to both pre-OIT treatment and placebo-treated subjects, studies have found improved QoL and less anxiety in subjects completing OIT [10–13]. In a prospective cohort study of parents of 191 food-allergic children 4–12 years of age undergoing OIT, QoL improved significantly upon reaching OIT maintenance on multiple dimensions including emotional impact (*p* = 0.001), food anxiety (*p* < 0.001), social and dietary limitation (*p* < 0.001), and global score (*p* < 0.001) [13].

The other benefit from OIT over time is to promote sustained tolerance to the allergen. Compared to placebo, OIT increases the chance of achieving sustained unresponsiveness to the allergen by seven-fold [5]. This benefit is most evident in the infant/toddler population when there is still a potential for outgrowing the allergy. The odds of achieving sustained unresponsiveness with OIT is inversely correlated with age and specific IgE levels, reaching up to 78% for peanut in infants and toddlers under 4 years of age [7]. An important finding from IMPACT–an international randomized controlled trial (RCT) assessing long-term outcomes of peanut OIT in peanut-allergic children 1–3 years of age–was sustained unresponsiveness being highly enhanced in the youngest toddlers with low baseline peanut sIgE (71% sustained unresponsiveness for screening age 12.0–23.9 months vs. 35% for age 24.0–35.9 months vs. 19% for age 36.0–47.9 months; *p* = 0.013) [9]. In this trial, the definition for sustained unresponsiveness was a very long period of treatment cessation (discontinuation of OIT for 26 weeks).

While older patients with severe allergy are not expected to achieve sustained unresponsiveness with OIT, they can still attain sustained benefits from the treatment. In fact, even if they do not tolerate the full OFC, studies have shown that the vast majority of patients do tolerate higher doses of allergen after discontinuing therapy compared to their OFCs at study entry [14]. This likely reflects protection from allergen-specific IgG4, which are produced as a result of OIT regardless of age.

The benefits of OIT have also been confirmed in real-world studies. The Canadian Preschool Peanut Oral Immunotherapy (CPP-OIT) project found that, of 117 toddlers (mean age = 26 months) who successfully completed peanut OIT and underwent a cumulative 4000-mg follow-up OFC, 78.6% had a negative challenge and 98.3% tolerated a cumulative dose of > 1000 mg [15]. Another recent Canadian real-world study found that low-dose sesame OIT (200 mg maintenance dose) was safe and led to the successful desensitization of 18 of 21 (85%) preschoolers with sesame allergy as measured on their exit OFC [16]. Only one patient (3.6%) required epinephrine during the build-up phase, and no patients required epinephrine in the maintenance phase.

It should be noted that the preschool-age population appears to have better outcomes with OIT than older children, with higher rates of desensitization and a lower risk of adverse events, including anaphylaxis [6, 17, 18].

### Safety of OIT

Overall, OIT is considered safe, although adverse reactions to food doses can occur. Mild-to-moderate cutaneous or gastrointestinal symptoms, such as local urticaria, oropharyngeal pruritus and abdominal pain, are common. Severe systemic reactions, such as anaphylaxis, are less common but can also occur. A systematic review and meta-analysis of 12 studies including primarily older children (median age across trials was 8.7 years) found a higher rate of anaphylaxis (16.5%) during peanut OIT compared with avoidance (2.7%) [19]. As mentioned earlier, side effects to OIT are more common in the older population [6, 9, 17, 18]. However, it is important to note that systematic reviews typically use the placebo arms of clinical trials to estimate safety risks with avoidance, but a more clinically relevant comparator is the real-world risk of avoidance as demonstrated in prospective data from a 3-year follow-up of children (median age at follow-up was 11.5 years) with double-blind, placebo-controlled OFC-confirmed peanut allergy [20]. What was most concerning from this real-world data was not only that 29% experienced severe symptoms, but moreover none of the reactions were treated with epinephrine.

In a meta-analysis by the Global Allergy and Asthma European Network (GA2LEN) Food Allergy Guideline Group that included 36 RCTs involving 2126 participants (primarily children), OIT was not associated with a significant increase in adverse or severe reactions in peanut allergy; however, an increase in mild adverse reactions (primarily oral pruritus and gastrointestinal pain) in cow’s milk and hen’s egg allergy was observed [5].

The safety of peanut and tree nut OIT in preschoolers has been established in the real-world Canadian population [21, 22]. Soller et al. administered peanut OIT to 270 preschoolers in the real-world setting and found treatment to be safe for the majority of patients, with 71.2% of reactions during build-up being mild, 0.2% of reactions being severe, 2.2% of reactions requiring epinephrine, and a low dropout rate (10%) [21]. Another real-world, multicentre analysis of tree nut OIT in 97 preschoolers found OIT to be safe and tolerable [22]. The majority of patients experienced mild-to-moderate reactions during the build-up phase (70.6%), 2% received epinephrine and no serious grade 3 or 4 reactions were reported. A recent Canadian real-world study found the safety of preschool peanut OIT or peanut OIT using a slower build-up schedule to be comparable to that of subcutaneous immunotherapy (SCIT) (used for the treatment of common allergic conditions resulting from environmental/aeroallergens–see *Allergen Immunotherapy* article in this supplement [23]) despite differences in OIT protocols used and age groups studied [24].

An important factor to consider prior to starting OIT is the risk of eosinophilic esophagitis (EoE; see article on *EoE* in this supplement [25]). The rate of EoE in OIT is estimated to be between 0.5 and 5% [26]. However, it is important to note that children with food allergies are already at risk of EoE at baseline, although the risk appears to be lower in infants and toddlers [21]. At present, it remains unclear whether OIT causes EoE, or rather “unmasks” it in patients who had pre-existing, undiagnosed esophageal eosinophilia [27–32]. Additional studies are required to further evaluate the relationship between OIT and EoE. Until then, a practical approach based on shared decision-making for continuing OIT when it has unmasked EoE can be considered [33].

It is also important to consider cofactors that alter immune homeostasis or allergen absorption, such as viral infections, fever, exercise, use of non-steroid anti-inflammatory drugs (NSAIDs), hormonal changes and evening ingestion/dosing, as these may play a role in triggering an adverse reaction to an OIT dose that is otherwise usually tolerated [34, 35]. In general, avoidance of physical activity for at least 1 h before and 3 h after intake of a food allergen is recommended [34]. In those with a fever or infectious condition, suspension or reduction of the OIT food dose for a few days is generally advised.

### Infant OIT for failed primary prevention

Primary prevention of food allergy has become an important public health goal (see *Primary Prevention of Food Allergy: Beyond Early Introduction* article in this supplement) [36]. It involves early introduction of allergenic foods in high-risk infants (i.e., those with mild to moderate atopic dermatitis, a family history of atopy in either or both parents, or those with one known food allergy) to reduce the risk of development of food allergy [37]. None of the current primary prevention guidelines provide detailed advice for managing infants who experience failed primary prevention [37–39], beyond the implication that it results in the need for avoidance of the allergenic food(s) and carrying an epinephrine autoinjector.

Although population-level data describes predictors of which infants are most likely to outgrow food allergy [40], clinically an allergist cannot reliably predict whether an infant is likely to outgrow food allergy as there will be outliers. At present, novel biomarkers for predicting which individual infants will outgrow their food allergy are either not precise enough or not yet ready for use outside the research setting [41].

In early 2017, a seminal study published by Vickery et al. demonstrated for the first time that peanut OIT was very effective in peanut-allergic preschoolers aged 9–36 months (n = 40) who were randomized to either low-dose (300 mg) or high-dose (3000 mg) peanut OIT over a median of 29 months (including a 10.5-month build-up period) [7]. The desensitization rate was 85% in the low-dose group and 76% in the high-dose group. Furthermore, 78% of toddlers achieved sustained unresponsiveness to peanut 4 weeks after discontinuing OIT and reintroducing peanut into the diet. Predominantly mild symptoms, such as abdominal pain, skin/oral pruritus, nausea, sneezing/congestion and hives, were reported and there was one moderate reaction requiring epinephrine [7].

A recent Canadian study comparing OIT in infants (aged < 12 months) versus non-infant preschoolers (aged 12–70 months) found OIT to be equally effective in both groups, yet safer in infants [42]. As evidence of how important the outcome of safety is to stakeholders, a recently published international Delphi consensus study (“COMFA”) of 778 participants from 52 countries (which included patients and caregivers) found that only two outcomes achieved consensus for inclusion as “core” outcomes in future food allergy clinical trials and observational studies: allergic symptoms (i.e., safety) and QoL [43, 44].

As discussed above, IMPACT found that the earlier OIT is started in childhood, the greater the likelihood of sustained unresponsiveness or tolerance [9]. Also, the earlier OIT is initiated, the more cost effective it may be [45], with the added benefit of improving QoL considerably earlier than if it is deferred until a child is older [46].

Based on personal preferences, families of infants failing primary prevention may still choose avoidance. However, in the current era of shared decision-making, families should be informed that this may result in a permanently missed opportunity to induce sustained unresponsiveness. They should be made aware of infant OIT as a management option for failed primary prevention [47] and referred to an allergist if they are interested in pursuing this form of therapy. In areas where access to an allergist is limited, e-consultations can be used to determine the best course of action while waiting for an in-person consultation.

### OIT guidelines

Several allergy organizations have released guidelines on OIT [48], but the most relevant for Canadian clinicians are those published by the European Academy of Allergy and Clinical Immunology (EAACI) [49] and the Canadian Society of Allergy and Clinical Immunology (CSACI) [2] (Table 2). Both the EAACI and CSACI guidelines emphasize the importance of shared decision-making with patients and caregivers before initiating, and during, OIT. OIT is considered a personalized treatment that needs to be adapted to the patient’s context, individual risks and benefits, goals and objectives for therapy, eating habits, experience, and motivation.

**Table 2:** European and Canadian guidelines for OIT

Guidelines	Country	Key points/recommendations
EAACI guidelines [49]	Europe	• OIT is a therapeutic option for inducing desensitization in children from 4 to 5 years of age with an IgE-mediated food allergy for cow’s milk, eggs, and peanut • During the escalation phase, dose increases to be carried out under medical supervision within an infrastructure capable of treating allergic emergencies • Need for informed consent before the initiation of OIT • Contraindications and unresolved points identified
CSACI guidelines [2]	Canada	• Recommendations for a patient-centered practical approach, analyzing 22 criteria divided into 5 domains (sociopolitical, population, clinical, organizational, economic) • OIT can be offered to all patients, including adults, for all foods and also in the event of multiple food allergy • The notion of the severity of the initial reaction should not be taken into account to contraindicate OIT • Uncontrolled asthma is an absolute contraindication to starting OIT • During the escalation phase, dose increases to be carried out under medical supervision in a structure capable of treating allergic emergencies, with 1 h of monitoring • OIT can be carried out with various products, including those for industrial consumption • Need for informed consent before initiation of OIT

*CSACI* Canadian Society of Allergy and Clinical Immunology; *EAACI* European Academy of Allergy and Clinical Immunology (EAACI); *OIT* oral immunotherapy

Adapted from Pouessel G, Lezmi G. 2023 [48]

The EAACI recommends waiting for the allergy to resolve naturally before starting OIT, and only recommends it from the age of 4–5 year. It should be noted, however, that the EAACI recommendations were published in 2018 before more recent evidence from randomized trials such as IMPACT [9] became available showing greater efficacy of OIT when started at earlier preschool ages. Also, the EAACI only recommends OIT for cow’s milk, egg and peanut allergy, and does not recommend OIT for adults [49]. In contrast, the CSACI recommends OIT for all foods and for all patients (children and adults) wishing to receive it, provided that there are no contraindications to therapy and that patients and caregivers have a clear understanding of individual risks and benefits [2].

According to both guidelines, uncontrolled asthma and pregnancy are absolute contraindications for OIT, while active severe atopic dermatitis, pre-existing EoE, heart disease, and the use of beta-blockers or angiotensin-converting enzyme (ACE) inhibitors are relative contraindications based on clinical judgement, provider expertise, and shared decision-making [2, 49]. Patient- or caregiver-specific contexts that may jeopardize the safe administration of therapy must also be assessed. For example, failure to adhere to the OIT protocol and attend regular appointments and/or the inability to recognize and treat severe reactions (e.g., reluctance to use epinephrine) constitute contraindications for OIT [2, 49].

Both the CSACI and EAACI guidelines specify that allergists practicing OIT must have expertise in this type of care as well as infrastructure that allows for the regular and personalized follow-up of patients, the performance of OFCs, and the management of anaphylaxis [2, 49]. Informed consent that clearly specifies the risks and benefits of OIT must be obtained before initiating therapy.

### Access to OIT in Canada

There are disparities in accessing OIT in Canada, which are further exacerbated by increasing demand, the lack of specialized care in both urban and rural areas, and a potentially inadequate billing system in some Canadian provinces [2].

A survey conducted in 2021 indicated that over 50% of Canadian allergists (52.2%) offer OIT, mainly for peanut allergy. However, significant barriers to expanding OIT practice were reported, including lack of efficacy data, lack of support staff and clinic space, and concerns about increased OFCs [50]. Clinicians not offering OIT cited concerns about safety, after-hours support, efficacy, medicolegal risk, and long-term practice implications as major obstacles. Qualitative assessment revealed concerns about practical challenges associated with OIT, the need for increased safety and efficacy data, and the desire for OIT guidelines and training. Access to OIT in underserved areas will require collaboration between different healthcare professionals.

### Milk and egg ladders: a modified form of OIT

Cow’s milk and egg are among the most common food allergies in young children. Although milk and egg allergies have historically been regarded to have a good prognosis, with many children outgrowing these allergies in childhood, evidence suggests that the rate of resolution may be slowing over time, with only 50% resolution by 5–6 years of age and increasing persistence of these allergies into adolescence or adulthood [51, 52]. A recent US population-level study found that Black and Hispanic patients, as well those with non-cutaneous symptoms, were less likely to outgrow cow’s milk allergy than White patients or those with cutaneous symptoms [53].

There is increasing recognition that a subset of children with egg and milk allergy may tolerate baked/processed forms of milk and egg. Observational studies have found that patients who incorporated baked products in their diet were more likely to outgrow their allergy [54, 55]. This has led to the hypothesis that baked products could increase the odds of developing tolerance to the raw food, while being a safer approach than OIT with the raw product [56]. However, it is critical to recognize that this has never been demonstrated in a randomized trial. Observational trials are at very high risk of bias since those patients who introduced baked goods are likely to be those with milder allergies.

**Fig. 2:**
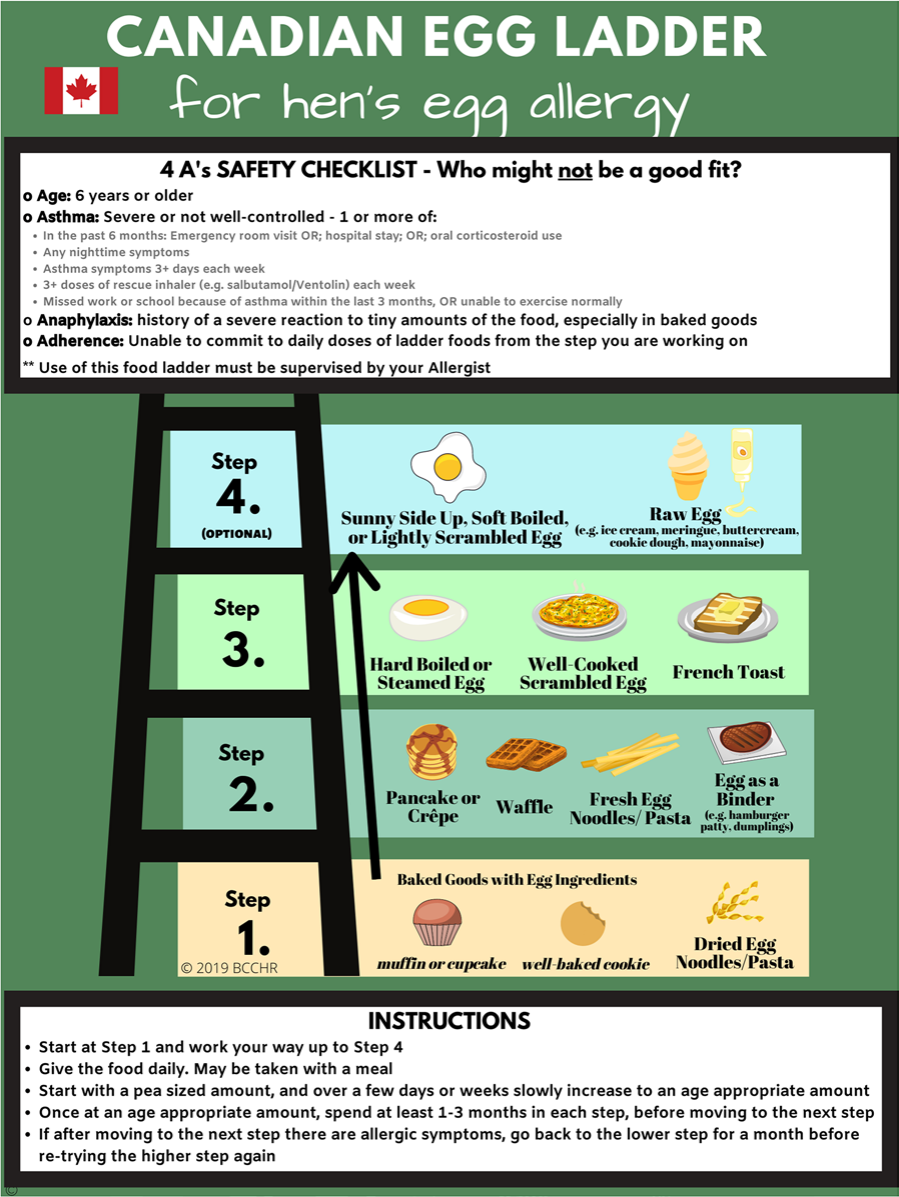
Canadian egg ladder [58]. Adapted from: Chomyn A, et al. Allergy Asthma Clin Immunol. 2021;17(1):83 [58]. Creative Commons license: https://creativecommons.org/licenses/by/4.0/ The image has been updated to include the 4 A’s safety checklist. The Canadian egg ladder is available at: https://www.bcchr.ca/sites/default/files/group-food-allergy-treatment/_canadian-egg-ladder_sept-15.png Accessed September 6, 2024

**Fig. 3:**
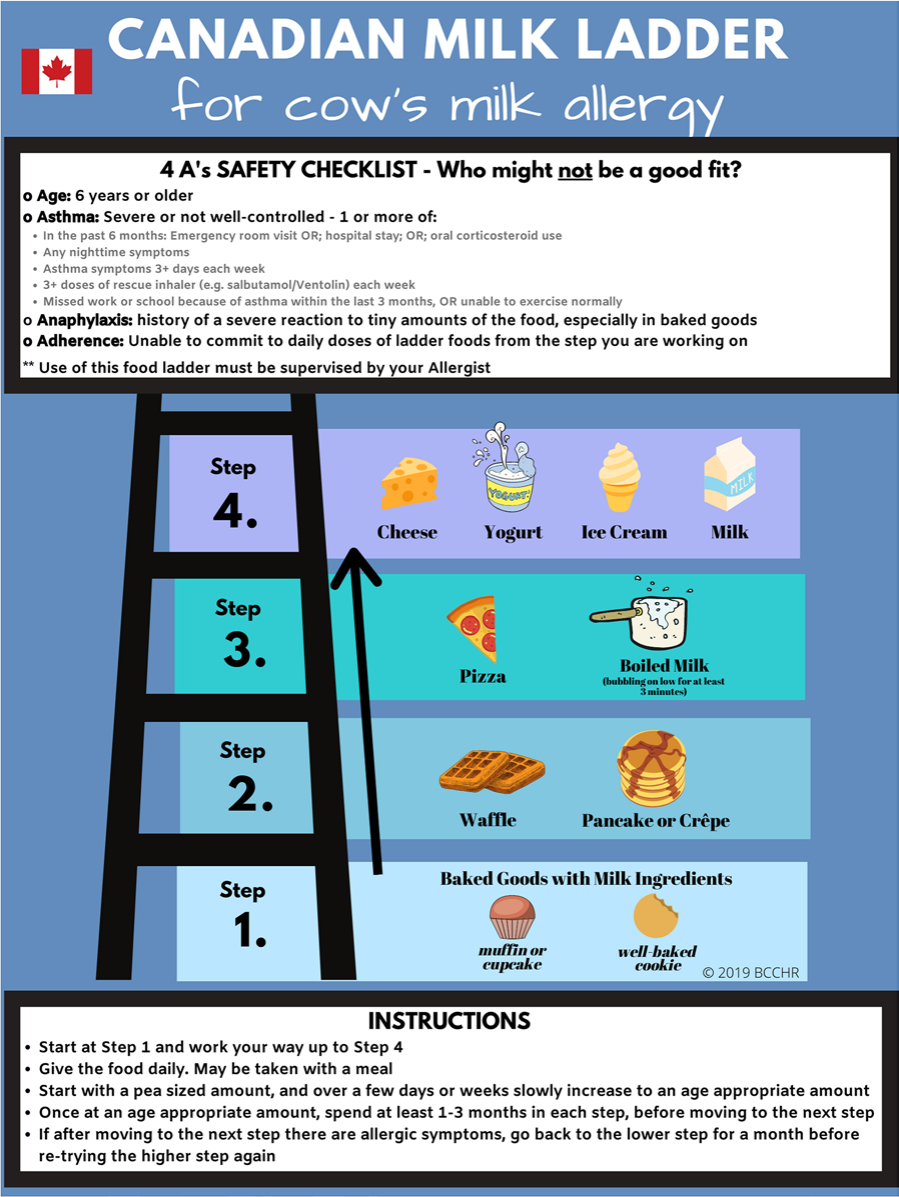
Canadian milk ladder [58]. Adapted from: Chomyn A, et al. Allergy Asthma Clin Immunol. 2021;17(1):83 [58]. Creative Commons license: https://creativecommons.org/licenses/by/4.0/ The image has been updated to include the 4 A’s safety checklist. The Canadian milk ladder is available at: https://www.bcchr.ca/sites/default/files/group-food-allergy-treatment/canadian-milk-ladder.png Accessed September 6, 2024

Food ladders were tools initially designed to guide patients with non-IgE-mediated food allergy through a home-based gradual, stepwise introduction of increasing allergenic forms of milk and egg (i.e., from extensively heated forms, such as baked goods [e.g., biscuits, muffins], to less processed products [e.g., yogurt or ice cream]) [57]. While originally designed to allow home reintroduction without the need for in-clinic OFCs, food ladders have more recently been proposed as a modified form of OIT to facilitate the development of natural tolerance to allergenic foods such as milk and egg [58] (see examples of food ladders in Figs. 2 and 3). A recent Canadian study found food ladders to be safe in children with IgE-mediated allergies to cow’s milk and/or egg, with participants tolerating a larger range of foods with food ladder use compared to baseline [59]. If food ladders are used for the purpose of facilitating the development of natural tolerance, they should be recognized as a modified form of OIT, carrying the same risks as traditional OIT. As such, food ladders should be administered by well-trained and experienced healthcare professionals with the necessary expertise in food allergy and anaphylaxis management, performance of OFCs, and careful selection of patients for food immunotherapy [60]. Failure to recognize that food ladders are being used as OIT can lead to a false sense of safety.

Appropriate patient selection for home-based egg and milk ladders is paramount. Experts have recently proposed a food ladder safety checklist to assist with patient selection using “4 A’s” based on available evidence for food ladders, including ***A***ge, active or poorly controlled ***A***sthma, history of ***A***naphylaxis, and ***A***dherence (see Figs. 2 and 3) [60]. Allergists may decline or delay offering food ladders while optimizing any modifiable factors, such as asthma, or may opt for an alternative dietary advancement therapy such as traditional OIT. Similar to traditional OIT, the decision to start a patient on a food ladder should be based on shared decision-making between the specialist, patient and caregiver, and therapy should be personalized to each individual patient/family.

### Key considerations for non-allergist clinicians

Pediatricians, family physicians and other healthcare professionals working in the pediatric and family practice settings can play a key role in identifying patients with IgE-mediated food allergies who may be appropriate for OIT and who could benefit from referral to an allergist.

There are no consensus criteria to select patients for whom OIT could be proposed. Several factors should be considered such as patient age (i.e., OIT is safer and more effective in younger children), the natural evolution of the food allergy, the presence of comorbidities that may be contraindications for OIT (e.g., severe uncontrolled asthma [absolute contraindication], EoE [relative contraindication]), the patient’s experience (e.g., the burden of food allergy and impact on QoL), and the patient’s/family’s ability to understand the risks and benefits of therapy, adhere to the treatment protocol and manage possible adverse reactions to therapy. As mentioned earlier, families of infants who have failed primary prevention of food allergy (see *Primary Prevention of Food Allergy: Beyond Early Introduction* article in this supplement [36]) should be provided with infant OIT as a management option [60] and should be referred to an allergist if they are interested in pursuing this form of therapy.

**Fig. 4:**
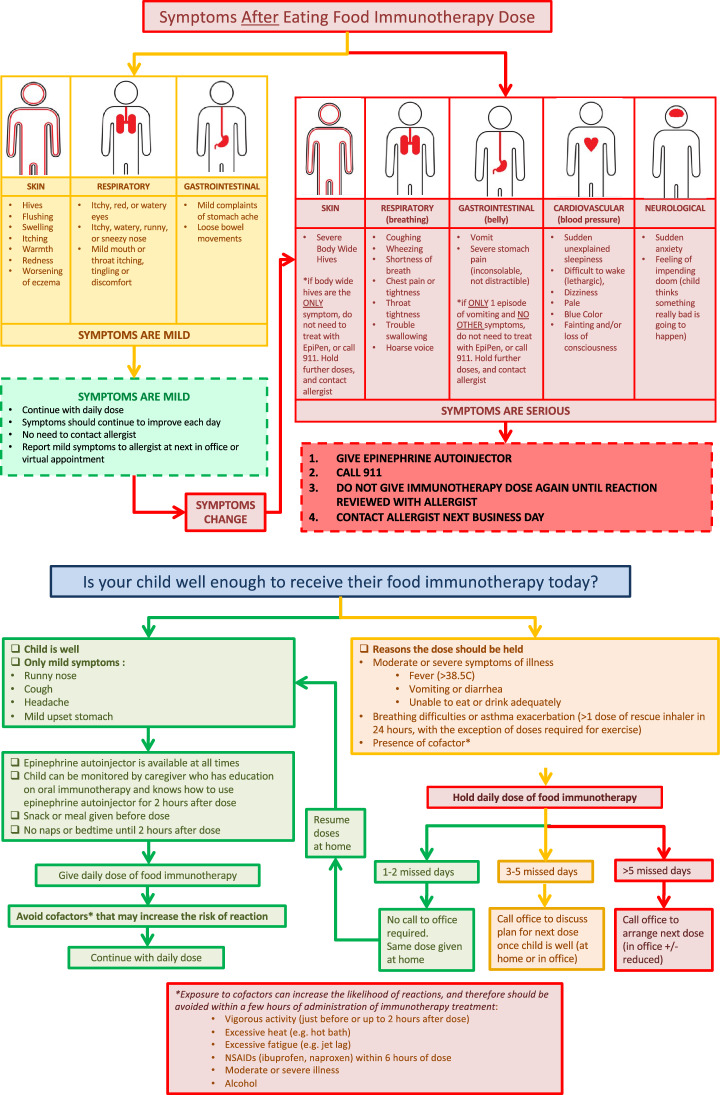
Flow sheet for parents–daily dose instructions and side effect management. Figure reproduced with permission from Dr. Lianne Soller on behalf of the Canadian Preschool Peanut Oral Immunotherapy (CPP-OIT) project

Pediatricians and family physicians can also play an important role in the prevention and management of side effects of OIT as they can often be the first point of contact for patients experiencing an adverse reaction to therapy (see Table 3 for management strategies) [61]. Children undergoing OIT and their parents should be made aware of the possible occurrence of adverse events and how to treat these events. It is therefore important for pediatricians and family physicians to reinforce education received by the allergist and to ensure that families are equipped with an epinephrine auto-injector as well as a clear plan or flow sheet on how to manage at-home reactions, when to hold/reduce OIT doses (e.g., during a viral illness) and when to administer epinephrine (see Fig. 4) [21].

**Table 3:** Management of patients on OIT by pediatricians and family physicians [61]

General management	
General advice	• Reinforce education received by allergist —Take the food dose after school/work (e.g., during dinner) —Do not take the dose when fasting —Avoid physical activity 1 h before and for 3 h after the food dose —Avoid NSAIDs
In the presence of co-factors (fever and viral infection, anti-inflammatory intake, gastrointestinal disease, physical activity, menstruation, etc.)	• Avoid co-factors if possible • Prescribe preventive non-sedating oral antihistamine • Transiently decrease daily dose (consult with allergist) • Temporarily discontinue OIT only in the case of severe acute illness–advise allergist
**Allergic side effects**	
Local (oral pruritus or edema, peri-oral urticaria) or mild systemic IgE-mediated symptoms (generalized urticaria, angioedema without dyspnea)	• Non-sedating oral antihistamine treatment in case of mild symptoms • Continue with the same dose if possible, with preventive non-sedating antihistamine for several days if needed • Determine if cofactor may be responsible for the reaction
Anaphylaxis (acute onset of hypotension or bronchospasm or laryngeal involvement up to several hours after allergen ingestion, even in absence of skin involvement)	• IM epinephrine ± SABA (salbutamol) in case of bronchospasm, and medical monitoring • Consider decreasing OIT dose (consult with allergist) and prescribe preventive non-sedating oral antihistamine • Determine if cofactor present; in absence of cofactor, decrease the food OIT dose • Advise allergist of the anaphylaxis
Non-IgE-mediated reactions (mainly suspicion of EoE: recurrent nausea, emesis, abdominal pain and refusal to eat in young children, dysphagia in older children and adolescents)	• Gastrointestinal expert advice needed • Advise the allergist

*EoE* eosinophilic esophagitis; *IM* intramuscular; *IgE* immunoglobulin-E; *NSAIDs* nonsteroidal anti-inflammatory drugs; *SABA* short-acting beta2-agonist; *OIT* oral immunotherapy

Reproduced from Sabouraud M, Biermé P, Andre-Gomez SA, Villard-Truc F, Corréard AK, Garnier L, et al. Oral immunotherapy in food allergies: A practical update for pediatricians. Arch Pediatr. 2021;28(4):319–324. Copyright ^©^ 2021 Elsevier Masson SAS. All rights reserved

In cases of acute reactions, a possible error by clinicians in the emergency department is to recommend full discontinuation of OIT doses while waiting to see the allergist. Ideally, doses should be reduced but not stopped since this can lead to loss of protection.

Finally, in regions with no access to an allergist, primary-care providers should seek partnership with allergists from urban centers to establish local protocols that allow for the early initiation of OIT in their patients.

### Future/emerging therapies

In addition to the expanding practice of OIT, there are other emerging therapies for the treatment of food allergy including sublingual immunotherapy (SLIT) and epicutaneous immunotherapy (EPIT). SLIT and EPIT consist of daily application of very small amounts of the allergen under the tongue with dissolvable tablets or liquid allergen extracts (SLIT) or on the skin with a patch device (EPIT). OIT has been the most studied form of food allergy treatment to date but, as side effects with OIT are common, SLIT and EPIT have been proposed as safer routes of administration with fewer adverse reactions. A recent placebo-controlled RCT found clinically significant desensitization with peanut SLIT compared to placebo in children aged 1–4 years with peanut allergy [62]. Side effects were similar between the SLIT and placebo groups, and no reactions required epinephrine. While SLIT may be safer than OIT (i.e., less side effects and less risk of epinephrine use), its efficacy may be slightly lower. In the above-mentioned RCT, 60% and 48% of peanut SLIT participants demonstrated desensitization and sustained unresponsiveness, respectively, compared to no placebo participants [62]. Higher rates of desensitization and sustained unresponsiveness are typically seen with OIT. Recently, an initial phase of multi-food SLIT using fresh food solutions (given the lack of Health Canada approved products) was used in a real-world Canadian study as a means to bypass OIT buildup in older children and adolescents for whom OIT is considered to be of higher risk [63]. None of the patients had severe reactions during SLIT, and 70% completed the low-dose OFC without any symptoms, allowing them to bypass OIT build-up and go directly from SLIT to OIT maintenance.

Emerging evidence also suggests that EPIT is safe and effective for the treatment of food allergy, as demonstrated in a recent phase 3 RCT that found 12 months of EPIT to be superior to placebo in desensitizing peanut-allergic children aged 1–3 years to peanut and a low rate of treatment-related anaphylaxis (1.6% in the EPIT group and none in the placebo group) [64]. Biologics, such as omalizumab (an anti-IgE monoclonal antibody that was first approved to treat allergic asthma), are also being studied as adjuvant treatment during OIT to reduce the risk of severe reactions [48, 65]. CSACI guidelines suggest that omalizumab as an off-label adjunct to OIT could be considered in more challenging cases to decrease the risk of anaphylaxis and accelerate treatment [2]. In February 2024, the Food and Drug Administration (FDA) approved omalizumab for reducing allergic reactions, including anaphylaxis, resulting from accidental exposure to one or more foods in individuals ≥ 1 year of age with IgE-mediated food allergies [66]. This approval was based on the initial stage of the OUtMATCH trial which showed that a 16-to-20-week course of omalizumab increased the amount of peanut, tree nuts, egg, milk and wheat that multi-food allergic children could consume without a moderate or severe allergic reaction [67]. The second stage of OUtMATCH will compare omalizumab monotherapy to omalizumab combined with OIT in patients with multi-food allergies [68]. Health Canada has not approved omalizumab for treatment of food allergy.

Currently, most OIT is supervised by an allergist in clinic, but there are reports of home-based OIT as an option for select patients. A case series reported by Chua et al. showed that home-based OIT could be offered to low-risk preschoolers during the coronavirus disease (COVID-19) pandemic [69]. Nine preschoolers with a history of mild allergic reactions to peanut underwent home-based peanut OIT. Eight (88.9%) completed the build-up phase at home in 11–28 weeks, tolerating a daily maintenance dose of 320 mg of peanut protein. Symptoms were common, but mild to moderate: six patients (75.0%) reported urticaria, three (33.3%) reported gastrointestinal tract symptoms, and one (14.3%) reported oral pruritis. None of the patients developed anaphylaxis, required epinephrine, or attended emergency services related to OIT. One or two virtual follow-up visits were completed per patient during the build-up phase.

## Conclusions

OIT is a safe and effective treatment option for IgE‐mediated food allergy. Current Canadian, patient-centred, clinical practice guidelines recommend OIT for patients with IgE-mediated food allergy wishing to receive it, provided that there are no contraindications to therapy and that patients and caregivers clearly understand the benefits and risks of therapy. Shared decision-making between the patient, family and allergist are imperative before initiating OIT.

OIT is likely to become a more routine, standard therapeutic option in food allergy management in the future, but strategies are needed to address disparities in access to this form of therapy across Canada. Further research is needed on the long-term efficacy of OIT, and international consensus on safety reporting for OIT is required to facilitate the development of personalized protocols that improve safety outcomes.

## Data Availability

Data sharing not applicable to this article as no datasets were generated or analyzed during the development of this review.

## Abbreviations

ACE: Angiotensin-converting enzyme
CI: Confidence interval
CPP-OIT: Canadian Preschool Peanut Oral Immunotherapy
CSACI: Canadian Society of Allergy and Immunology
COVID-19: Coronavirus disease
EAACI: European Academy of Allergy and Clinical Immunology
EoE: Eosinophilic esophagitis
EPIT: Epicutaneous immunotherapy
GA2LEN: Global Allergy and Asthma European Network
IgE: Immunoglobulin E
IgG4: Immunoglobulin G4
NSAIDs: Non-steroid anti-inflammatory drugs
OFC: Oral food challenge
OIT: Oral immunotherapy
QoL: Quality of life
RCT: Randomized controlled trial
RR: Risk ratio
SABA: Short-acting beta_2_-agonist
SLIT: Sublingual immunotherapy
